# Peripheral Facial Paralysis as a Sentinel Symptom of Early Lyme Neuroborreliosis: A Diagnostic Challenge

**DOI:** 10.7759/cureus.96982

**Published:** 2025-11-16

**Authors:** Mohamed Bouallou, Achraf Amine Sbai, Drissia Benfadil, Azzedine Lachkar, Fahd El Ayoubi El Idrissi

**Affiliations:** 1 Department of Otolaryngology - Head and Neck Surgery, Mohammed VI University Hospital Center, Oujda, MAR

**Keywords:** borrelia burgdorferi infection, doxycycline treatment, facial nerve paralysis, lyme disease, neuroborreliosis

## Abstract

Lyme disease, while well recognized in many regions of the Northern Hemisphere, is generally considered uncommon in Morocco. While erythema migrans represents its hallmark cutaneous manifestation, neuroborreliosis remains a challenging diagnosis, particularly when presenting as isolated peripheral facial paralysis, often mimicking idiopathic Bell’s palsy.

We report the case of a 55-year-old man presenting with fever followed six weeks later by left-sided peripheral facial paralysis. Neurological examination revealed stage IV facial palsy according to the House-Brackmann classification. Radiological assessment, including computed tomography of the brain and temporal bones as well as magnetic resonance imaging, revealed no abnormalities. Laboratory investigations excluded viral and autoimmune causes. *Borrelia burgdorferi* immunoglobulin M and immunoglobulin G (IgG) antibodies were detected using the enzyme-linked immunosorbent assay method. Serum Western blot testing confirmed positivity with 8 of 10 IgG bands. Cerebrospinal fluid analysis revealed pleocytosis, with normal protein and glucose levels. A diagnosis of Lyme neuroborreliosis was established. The patient received doxycycline at a dose of 200 mg daily for six weeks, in combination with facial physiotherapy, and was subsequently discharged after achieving full clinical recovery, characterized by complete resolution of the facial paralysis, with normalization of inflammatory biomarkers.

This case underlines the importance of considering Lyme neuroborreliosis in the differential diagnosis of facial paralysis, even in non-endemic regions such as Morocco, and highlights the role of comprehensive clinical, radiological, and serological evaluation in establishing an accurate diagnosis.

## Introduction

Lyme disease, or Lyme borreliosis, classically begins with erythema migrans, a pathognomonic cutaneous manifestation at the site of inoculation. Peripheral facial palsy, however, is a rare neurological complication of the disease. With subsequent hematogenous dissemination, the disease may give rise to neurological, cardiac, or rheumatologic complications. Lyme disease is a systemic, non-contagious infection caused by the spirochete *Borrelia burgdorferi* and constitutes the most common tick-borne illness in both the United States and Europe, with an estimated annual incidence of 450,000 cases in the United States and around 65,000 cases in Europe [1]. Its occurrence in Morocco is rare, and the precise incidence remains undetermined.

*Borrelia burgdorferi* can affect the nervous system through diverse mechanisms, often creating considerable diagnostic challenges and clinical uncertainty. Lyme neuroborreliosis involves both the peripheral and central nervous systems. The clinical manifestations of Lyme disease are stage-dependent, with neurological involvement (Lyme neuroborreliosis) documented in up to 12% of cases [2]. The facial nerve, one of the principal cranial nerves, exhibits a highly intricate anatomy and course, which underlies the wide spectrum of clinical manifestations that may arise from its dysfunction. It is a mixed nerve, comprising sensory (afferent) fibers as well as parasympathetic (secretomotor) fibers. The most common cause of unilateral facial nerve injury is idiopathic peripheral facial paralysis, known as Bell’s palsy, which accounts for approximately 75% of all facial nerve palsies and is widely believed to have a viral etiology [3]. *Borrelia burgdorferi* infection has recently gained increasing recognition as a potential etiology of facial nerve paralysis, particularly when the paralysis occurs in association with fever. Nevertheless, additional neurological complications include cerebrovascular events resulting from post-infectious arteriosclerosis, embolism, vasculitis, hemorrhage, and cerebral venous sinus thrombosis [4].

The risk is heightened during May and October, when environmental temperatures fall within the optimal range for tick activity [5]. The early localized stage, occurring 3 to 30 days after the tick bite, is typified by erythema migrans and may be associated with flu-like symptoms, polyneuropathy, and diffuse arthralgias, which typically resolve spontaneously within approximately one month [6]. The secondary stage develops several weeks to months post-infection and is characterized by multisystem involvement, predominantly affecting the musculoskeletal, nervous, and cardiovascular systems. The late stage ensues six months to several years following the onset of infection, irrespective of prior treatment, and is marked by the chronic evolution of neurological lesions [5].

## Case presentation

**Figure 1:**
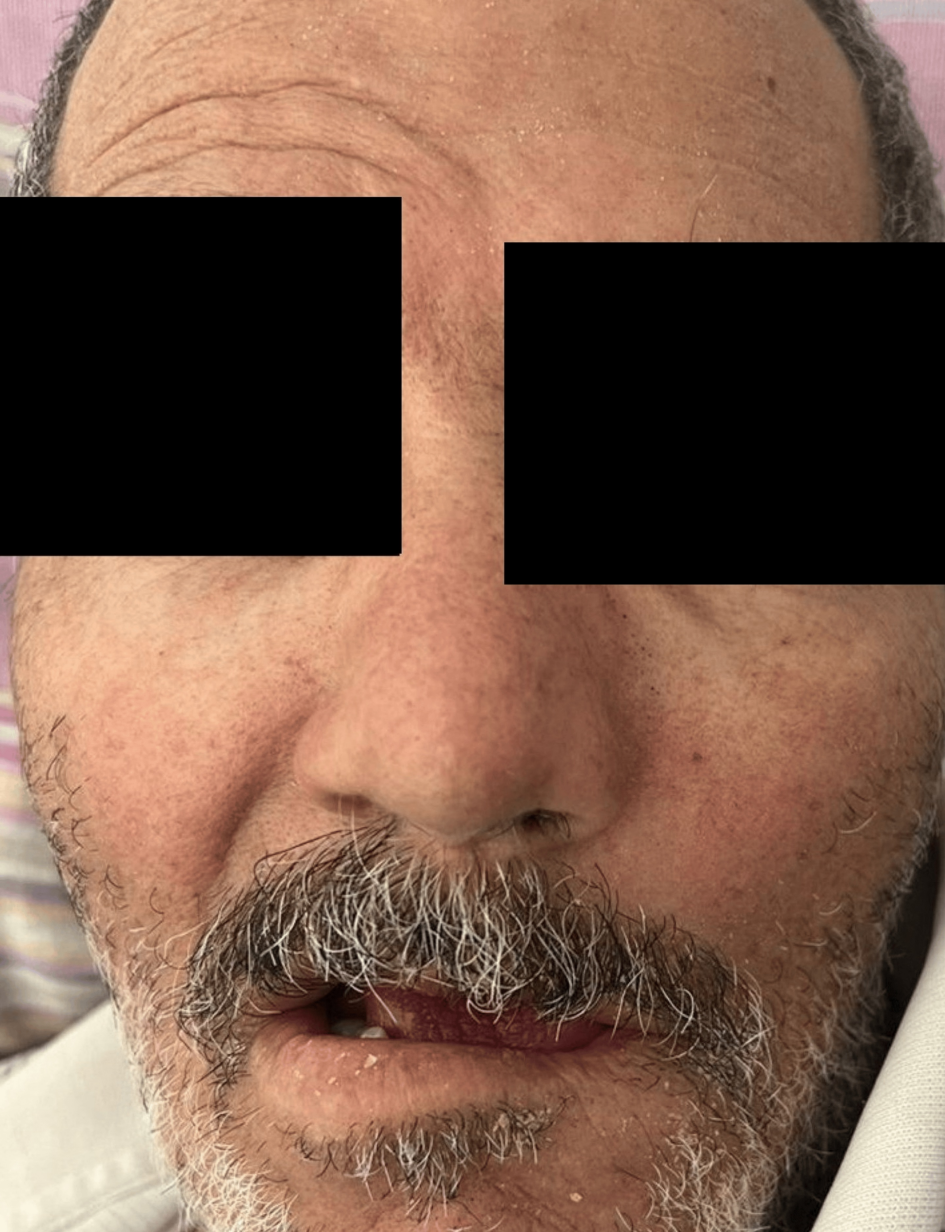
Grade IV facial paralysis according to the House–Brackmann classification

We report the case of a 55-year-old male patient residing in an urban area with no significant past medical history. The patient’s illness began 17 days prior with the onset of fever, which initially responded well to an antipyretic. Six weeks later, he developed left-sided facial paralysis, with loss of taste sensation in the anterior two‑thirds of the tongue (Figure 1). He subsequently consulted a general practitioner. At the time of the consultation, the hypothesis of Bell's palsy was suggested, and prednisolone 20 mg per day was started for a period of five days. Due to the lack of improvement and persistent left facial paralysis, he was referred to our department for management. Additionally, the patient reported a tick bite in the left temporal region, which he personally removed. There was no history of associated headache, vomiting, loss of consciousness, localized or radiating neck pain, myalgia, stiffness, respiratory distress, abdominal pain, arthralgia, or recent travel.

On general examination, the patient was conscious and fully oriented (GCS 15/15). The patient’s body temperature was 38.3 degrees Celsius. The respiratory rate and heart rate were 20 breaths per minute and 107 beats per minute, respectively, while blood pressure measured 129/77 millimeters of mercury. Dermatological examination revealed no evidence of erythema, particularly in the temporal region. Respiratory and cardiovascular examinations were unremarkable, and the abdominal examination revealed no abnormalities. Neurological examination revealed effacement of the left nasolabial fold and ptosis of the left oral commissure. The facial paralysis was graded as stage IV according to the House-Brackmann classification. Examination of the other cranial nerves was unremarkable. Motor strength in both upper and lower limbs was preserved, and deep tendon reflexes were normoactive. Otologic examinations were unremarkable. Cervical examination revealed no lymphadenopathy.

Cerebral and temporal bone imaging, including both CT and MRI, revealed no abnormalities. As part of the baseline evaluation, a complete blood count was obtained and was unremarkable (Table 1). Laboratory investigations, including electrolyte analysis, hepatic and renal function tests, and inflammatory markers, were within normal limits except for an elevated C-reactive protein level of 17.5 mg/dL (Table 2).

**Table 1:** Complete blood count MCV: Mean Corpuscular Volume; MCH: Mean Corpuscular Hemoglobin. The analysis revealed values entirely falling within normal reference limits.

Test	Value	Reference range
Leukocytes (/µL)	9700	4000-10000
Erythrocytes (*10^6^/µL)	5.1	5-5.5
Hemoglobin (g/dL)	13.7	13-18
Hematocrit (%)	41	40-54
Erythrocyte MCV (fL)	87	80-98
Erythrocyte MCH (pg)	29,1	27-32
Platelets (/µL)	221000	150000-400000
Neutrophils (/µL)	3300	1500-7000
Lymphocytes (/µL)	1300	1000-4000
Monocytes (/µL)	300	200-800
Basophils (/µL)	10	0-200

**Table 2:** Baseline laboratory investigations: renal, hepatic, electrolyte, and inflammatory parameters ALAT: Alanine Aminotransferase; ASAT: Aspartate Aminotransferase

Test	Value	Reference range
Urea (g/L)	0.37	0.15-0.45
Creatinine (mg/L)	10,3	7.2-12.5
Albumin (g/L)	43	35-50
ASAT (UI/L)	16	5-34
ALAT (UI/L)	11	0-55
Sodium (mEq/L)	141	136-145
Potassium (mEq/L)	4.2	3.5-5.1
Corrected calcium (mg/L)	93	85-102
Chloride (mEq/L)	105	98-107
C‑reactive protein (CRP) (mg/L)	17.5	0-5
Procalcitonin (ng/mL)	0.07	<0.1

Serological and molecular investigations yielded unremarkable findings, thereby excluding the principal viral, bacterial, and parasitic infections (Table 3).

**Table 3:** Serological and PCR analysis of potential infectious etiologies

Pathogen / Test	Result
Rubella IgM	Negative
Rubella IgG	Positive
Toxoplasma	Negative
Cytomegalovirus (CMV)	Negative
Hepatitis A	Negative
Hepatitis B	Negative
Hepatitis C	Negative
Herpes simplex virus (HSV) IgM/IgG	Negative
Varicella zoster virus (VZV) IgM/IgG	Negative
Epstein-Barr virus (EBV) IgM/IgG	Negative
PCR HSV 1 & 2	Negative

*Borrelia burgdorferi* IgM and IgG antibodies were detected by the ELISA method. Western blot analysis for *Borrelia burgdorferi* confirmed the presence of two of three positive IgM bands and eight of 10 IgG bands. Cerebrospinal fluid analysis showed a white cell count of 37 cells/µL with 85% lymphocytes, consistent with mild lymphocytic pleocytosis, with protein levels (55 mg/dL; reference range: 15-67 mg/dL) and glucose concentration (49 mg/dL; reference range: 50-80 mg/dL) within the normal range.

Based on these findings, a diagnosis of Lyme neuroborreliosis was established. After multidisciplinary discussion, a therapeutic decision was made to initiate doxycycline at a daily dose of 200 mg for six weeks, in combination with facial physiotherapy. The patient showed rapid and sustained improvement, with complete clinical recovery characterized by resolution of facial paralysis and normalization of inflammatory markers.

At the nine-month follow-up, the patient exhibited a stable and complete recovery of facial nerve function (House-Brackmann grade I), with no residual neurological deficits or recurrence, confirming the long-term effectiveness of treatment and an excellent functional outcome in our case.

## Discussion

Lyme disease is a zoonotic infection induced by the spirochete *Borrelia burgdorferi*. The etiologic agent was first identified by Willy Burgdorfer in 1982, following its isolation from *Ixodes dammini* ticks, a species commonly found on North American deer [7]. Borreliella burgdorferi invades the skin at the site of tick attachment, typically triggering the characteristic erythema migrans rash. Perhaps up to 60% of patients may fail to demonstrate it, and certain cutaneous diseases may act as false positives. Following inoculation, the pathogen may disseminate to the skin, nervous system, heart, and joints. However, patients may sometimes present at the stage of neuroborreliosis. Furthermore, most of the published literature consists of case reports involving adults residing in rural areas [8].

Lyme neuroborreliosis affects nearly 15% of untreated individuals in the United States [9], while its incidence in Morocco remains undetermined. In Europe, Lyme Neuroborreliosis represents the most frequent extracutaneous manifestation of Lyme disease, with peripheral facial palsy being the predominant clinical presentation [10,11]. Morocco is not recognized as an endemic region for Lyme disease, and no systematic epidemiological data have been reported. Indeed, involvement of other cranial nerves can occur in neuroborreliosis.

The pathophysiology of facial nerve paralysis in Lyme disease is multifactorial. In cases of *Borrelia burgdorferi* infection, two principal mechanisms are thought to contribute to the development of peripheral neuropathies. The first involves direct spirochetal interaction with neural cells, resulting in structural and functional nerve damage. The second is mediated by the host immune response, in which T- and B-cell autoreactivity against endogenous neural antigens contributes to inflammatory demyelination and further neuronal injury [3,12]. In our patient, the facial paralysis is likely attributable to direct spirochetal involvement of the temporal branches of the facial nerve.

Clinically, Lyme disease progresses through distinct stages: the early localized stage, most commonly presenting as erythema migrans in at least 80% of patients, followed by early disseminated and late stages [13]. The characteristic erythema migrans lesion appears at the tick bite site, usually within 7-14 days post-exposure, with an incubation period ranging from 2 to 28 days. It is characterized by a slowly enlarging, oval or round, flat erythematous skin lesion [1]. During the summer months, patients may additionally exhibit nonspecific systemic symptoms, including headache, fever, chills, myalgia, and arthralgia, even in the absence of erythema migrans [14]. In the present case, facial paralysis constituted the sole reason for consultation; indeed, clinical examination did not reveal the presence of erythema migrans.

In regions where Lyme disease is non-endemic, the differential diagnosis of peripheral facial palsy includes idiopathic (Bell’s) palsy, viral neuropathies, particularly herpes simplex and varicella-zoster viruses; autoimmune or inflammatory neuropathies, and compressive lesions along the intracranial, intratemporal, or extracranial course of the facial nerve. In our patient, careful clinical and semiological assessment, together with normal viral serologies, demonstrated isolated peripheral facial paralysis without involvement of other cranial nerves. The combination of unremarkable neuroimaging of the temporal bone and brain, elevated C-reactive protein, a documented history of a recent tick bite, prompted consideration of an infectious etiology. Subsequent targeted Borrelia serology was positive, and cerebrospinal fluid pleocytosis confirming the diagnosis of Lyme neuroborreliosis and underscoring the importance of integrating clinical examination, serological testing, and imaging in the evaluation of atypical facial nerve palsy.

Lyme neuroborreliosis is classified into early and late manifestations and can involve either the central nervous system or the peripheral nervous system. The diagnosis of Lyme neuroborreliosis should be based on an integration of clinical and laboratory findings, as outlined by the American Academy of Neurology and the European Federation of Neurological Societies [1]. Approximately 15% of patients with untreated erythema migrans go on to develop early Lyme neuroborreliosis, with a median of 4 week between the initial skin lesion and the onset of neurological manifestations [15]. Notably, in our patient, peripheral facial paralysis developed six weeks after the tick bite.

Cranial neuropathies, particularly peripheral facial palsy, along with lymphocytic meningitis and radiculoneuritis, constitute the predominant neurological manifestations of Lyme neuroborreliosis. Although Lyme disease is considered rare in many regions, several reports from non-endemic countries have documented similar presentations. Notably, cases from India and Belgium have described unilateral or bilateral facial nerve involvement associated with positive Borrelia serology and, in some instances, cerebrospinal fluid pleocytosis [5,15].

In our patient, neurological examination and cerebrospinal fluid analysis demonstrated lymphocytic pleocytosis, accompanied by elevated C-reactive protein levels and positive *Borrelia burgdorferi* serology. These findings collectively fulfilled the established diagnostic criteria for Lyme neuroborreliosis. Indeed, patients with early Lyme neuroborreliosis presenting with facial palsy are frequently misdiagnosed as having Bell’s palsy [1]. Our patient was initially treated as Bell’s palsy; the lack of improvement in her symptoms prompted consultation at our department.

Management of Lyme neuroborreliosis is primarily guided by disease stage and clinical manifestations. Currently, beta-lactam antibiotics and doxycycline are the most commonly used treatment options. Kortela et al. compared treatment with intravenous ceftriaxone to oral doxycycline: With respect to the primary outcome of residual neurological symptoms at 12 months post-treatment, pooled estimates revealed no statistically significant difference between β-lactam antibiotics and doxycycline [16]. Furthermore, to assess the efficacy of extended versus conventional antibiotic treatment, a systematic review by Dersch et al. concluded that there was no significant difference between two- and six-week courses of oral doxycycline at 200 mg daily [17]. In our case, doxycycline was administered at a daily dose of 200 mg for six weeks, at the end of which a significant regression of the facial paralysis was observed.

## Conclusions

This case highlights the importance of considering Lyme neuroborreliosis in patients with atypical or refractory facial paralysis, even in the absence of erythema migrans. Early recognition is crucial to avoid misdiagnosis, prevent unnecessary corticosteroid exposure, and initiate appropriate antibiotic therapy. Raising awareness among otolaryngologists and general practitioners is essential, particularly in regions where Lyme disease is rare, such as Morocco.
